# PEBA/PDMS Composite Multilayer Hollow Fiber Membranes for the Selective Separation of Butanol by Pervaporation

**DOI:** 10.3390/membranes12101007

**Published:** 2022-10-17

**Authors:** Carla Arregoitia-Sarabia, Daniel González-Revuelta, Marcos Fallanza, Alfredo Ortiz, Daniel Gorri

**Affiliations:** Departamento de Ingenierías Química y Biomolecular, Universidad de Cantabria, Av. de los Castros s/n, 39005 Santander, Spain

**Keywords:** hydrophobic pervaporation, dip-coating, selective membranes, concentration polarization, biobutanol recovery

## Abstract

The growing interest in the production of biofuels has motivated numerous studies on separation techniques that allow the separation/concentration of organics produced by fermentation, improving productivity and performance. In this work, the preparation and characterization of new butanol-selective membranes was reported. The prepared membranes had a hollow fiber configuration and consisted of two dense selective layers: a first layer of PEBA and a second (outer) layer of PDMS. The membranes were tested to evaluate their separation performance in the selective removal of organics from a synthetic ABE solution. Membranes with various thicknesses were prepared in order to evaluate the effect of the PDMS protective layer on permeant fluxes and membrane selectivity. The mass transport phenomena in the pervaporation process were characterized using a resistances-in-series model. The experimental results showed that PEBA as the material of the dense separating layer is the most favorable in terms of selectivity towards butanol with respect to the other components of the feed stream. The addition of a protective layer of PDMS allows the sealing of possible pinholes; however, its thickness should be kept as small as possible since permeation fluxes decrease with increasing thickness of PDMS and this material also has greater selectivity towards acetone compared to other feed components.

## 1. Introduction

In recent years, a series of initiatives have been carried out to support research and development that allow the replacement of fossil fuels with biofuels produced from renewable resources [1,2,3]. Among the various biofuels that have attracted attention, biobutanol stands out as it has, compared to ethanol, a higher energy density, lower miscibility with water, and lower vapor pressure. In addition, important advances have taken place in the production of biobutanol from different feedstocks [4,5]. As commented by Iyyappan et al. [6], a process involving cost-effective substrate and efficient biobutanol recovery methods could help with the implementation of the biobutanol production industry. Sugarcane bagasse, algal biomass, crude glycerol, and lignocellulosic biomass are considered potential cost-effective substrates for the production of butanol, which could replace glucose-based substrates.

Although, to a large extent, n-butanol is currently produced by chemical synthesis, there is a growing interest in its production by a biochemical route, mainly through the process known as ABE fermentation, in which, in addition to n-butanol, microorganisms also produce acetone and ethanol [7]. The biological production of butanol is specific to several Clostridia species. Among these, *Clostridium acetobutylicum* is considered the main species for biobutanol production; although, in recent years, other options have also aroused interest, such as the use of *Escherichia coli* or genetically modified organisms [6,8]. ABE fermentation by *C. acetobutylicum* takes place in two phases: an acidogenesis phase wherein the microbes mainly produce acetic acid and butyric acid, followed by a solventogenesis phase wherein the microbes mainly produce ABE compounds [9].

The industrial implementation of the fermentation process has faced several obstacles [10], including the inhibition of microorganisms by the butanol formed during fermentation. This phenomenon causes the ABE fermentation to stop when the concentration of the solvents produced is around 2 wt%, causing low utilization of the substrate and the costly recovery of butanol from diluted solutions. The obstacle due to the low solvent tolerance could be solved with the continuous butanol removal from the fermentation broth. Various separation techniques have been proposed and studied to remove products and increase the efficiency of the fermentation process, including gas-stripping, liquid–liquid extraction, and membrane technologies such as pervaporation and membrane distillation [11,12,13,14,15]. Some technical-economic studies indicate that the in situ product recovery (ISPR) from the ABE fermentation broth not only increases the productivity and the yield of ABE by eliminating the product inhibition, but it also reduces the energy consumption and the separation cost [16].

Among the separation techniques, pervaporation has attracted sustained interest from the scientific community in recent times. Pervaporation is a technique that allows the separation of liquid mixtures by permeating their components at different rates through a dense selective membrane, applying a certain vacuum on the downstream side of the membrane to establish the driving force for mass transfer. A series of advantages are attributed to pervaporation, such as high selectivity, low operating temperature, reasonable performance to cost ratio, possibility of modular design, and the absence of a separating agent that could cause product contamination [17,18]. Particularly, the low/moderate operating temperatures make pervaporation especially useful to work coupled with a bioreactor without harming the activity of microorganisms [7]. In addition, potential drawbacks associated with the use of membranes should be taken into account, such as membrane fouling, high equipment cost, and low/moderate productivity conditioned by permeation fluxes through the membrane [15,16].

For the case that interests us, which involves the selective removal of organic compounds from an aqueous solution, hydrophobic pervaporation membranes are used. Various polymeric materials for membranes have been tested for the separation of alcohols in the studies reported in the literature, among which poly(dimethylsiloxane) (PDMS) is the most used and, to a lesser extent, there are poly(ether-block-amide) (PEBA), poly(octylmethylsiloxane) (POMS), poly[1-(trimethylsilyl)-1-propyne] (PTMSP), polyurethane, and poly(vinylidene fluoride) (PVDF) [19,20,21]. In addition, in recent years, there has been an increasing interest in composite membranes formed with a polymer matrix with the addition of fillers such as zeolites [22,23], ZIF [24], MOF [25], and graphene oxide [26]. From all these polymer materials, PEBA has been especially selective towards butanol [27] due to its affinity for this compound that allows an appreciable solubility of butanol in the polymeric matrix, while the PEBA’s hydrophobic character limits the transport of water through the membrane.

Although most of the studies on pervaporation reported in the literature are carried out with flat sheet membranes, there is a growing interest in the development of membranes with a hollow fiber configuration that allow the construction of compact membrane modules with high membrane surface areas [28]. Fibers can be made as isotropic membranes with a uniformly dense structure [29], but they are preferably formed as a microporous structure with a dense selective layer on the outside or inside surface (anisotropic membranes) [30]. The dense surface layer can be either integral with the fiber or a separate layer coated on the porous support fiber. Given that our purpose is to obtain hollow fibers with a thin dense layer of PEBA, it was found in previous studies [31] that a suitable way of doing this is by depositing a thin layer of PEBA by dip-coating on a porous support of a suitable material, such as polypropylene. However, the aim of achieving PEBA dense layer thicknesses equal to or lower than 1 μm makes the presence of pinholes on the membrane surface more likely, causing selectivity losses. One way to repair the presence of pinholes is to cover the membrane with a second thin coating layer of a relatively permeable material such as silicone rubber to seal defects. Thus, a defect-free membrane would be obtained where the silicone rubber protective layer improves selectivity at the cost of a certain decrease in permeability.

Several examples of studies on multilayer membranes, including a protective layer of PDMS, are mentioned in the review papers by Dai et al. [32] and by Kujawski and col. [33]. Yahaya [34] reported a study on the separation of phenol from aqueous streams by PV using PDMS/PEBA two-layer hollow fiber membranes. From the pervaporation experiments, it was found that a significant improvement in the phenol/water separation factor and phenol flux was achieved with two-layer (PDMS/PEBA) membranes compared to that achieved using only the PDMS membrane. These results indicated that two-layer membranes combine the unique features of PDMS (exhibiting high permeability) and PEBA (exhibiting high permselectivity) to achieve this improvement in membrane performance. Jiang and Song [35] prepared polysulfone (outer layer)/Matrimid (inner) dual-layer hollow fiber PV membranes, applying them for tert-butanol dehydration. This work is a useful reference for mass transfer modeling since it uses a resistances-in-series model adapted to the cylindrical configuration of hollow fibers.

In the present work, a study about the preparation of multilayer membranes with hollow fiber configuration is reported. The fibers have two separating dense layers: a first layer of PEBA and a second (outer) layer of PDMS. The membranes were characterized in terms of their morphology and were subsequently tested to evaluate their separation performance in the selective removal of organics from a synthetic ABE solution. Membranes with various thicknesses of PDMS were prepared in order to evaluate the effect of the PDMS protective layer on permeant fluxes and membrane selectivity.

## 2. Materials and Methods

### 2.1. Materials

For the preparation of the composite HF membranes, Celgard X-20 polypropylene (PP) commercial HF membranes (supplied by Celanese) were used as support. These fibers had an internal diameter of 400 μm and a wall thickness of 30 μm, the porosity was equal to 40%, and the pores in the membrane were approximately 0.115 μm in diameter.

For the formation of the dense layers, the coating solutions were prepared with the following polymers: (a) Pebax 2533 (Hexanedioic acid, polymer with azacyclotridecan-2-one and .alpha.-hydro-.omega.-hydroxypoly(oxy-1,4-butanediyl)) (CAS No. 77402-38-1), which was kindly supplied by Arkema, France; (b) PDMS (Sylgard 184 silicone elastomer kit) was supplied by Dow Corning Company. n-Hexane (analytical grade, Honeywell) was used as the solvent for PDMS.

The aqueous feed solution for the PV experiments was prepared by mixing n-butanol (Merck), ethanol (Merck), and acetone (Riedel-de Haën) with ultrapure water Milli Q obtained from a Merck-Millipore system (supplied by Merck KGaA, Darmstadt, Germany). The acetone/butanol/ethanol content in the feed solution was 1:2:1 wt%. All of the materials were of analytical grade and were used without further purification.

### 2.2. Fabrication of Multilayer Hollow Fibers

As previously indicated, the purpose of this work was the preparation of multilayer membranes with hollow fiber configuration, incorporating two separating dense layers: a first layer of PEBA and a second (outer) layer of PDMS. Since both PEBA and PDMS polymers are rubbery materials, the deposition of two thin dense layers can be adequately carried out by dip-coating. The fabrication of ultrathin skin layer hollow fiber membranes implies a certain complexity in terms of the operating variables to be taken into account, as pointed out by Chung and col. [36]. Here, the dip-coating procedure consisted of immersing the commercial fibers in the coating solution for few seconds (3–5 s) to allow a thin film formation on the outer phase of the fibers. Covering of the ends of the fibers was previously performed to prevent the dip-coating solution from entering the fiber lumen.

The first layer was placed on the outside of the support by the dip-coating of a polymeric solution containing PEBA, following the procedure described in our previous work [31]. The coating solution used was prepared with a concentration of 2 wt% of PEBA in n-butanol, keeping it under stirring at 70 °C for 24 h. Then, it was left to rest for natural degasification and cooling for 24 h at room temperature. The viscosity was measured at room temperature using a rotational viscometer (Model Alpha Series L, Fungilab S.A., Spain), obtaining a value of 7.5 cP. The procedure that we followed allowed us to obtain a dense layer thickness of about 1.6 μm, as measured by SEM analysis. After depositing the PEBA layer, the fibers were left to stand for 1–2 days at room temperature for drying.

The second dense layer of the HF membranes was obtained by dip-coating with a PDMS coating solution. In all cases, the same PP support was used, and the dense layer thickness of PEBA was 1.6 μm. The PDMS polymer solution prepared from the two-component kit was slowly diluted in n-hexane to prepare various coating solutions, depending on the thickness of the dense layer to be obtained. The viscosity of the mixture was measured at room temperature using a rotational viscometer. Finally, the dip-coating of the hollow fibers was conducted by immersing a fiber for a few seconds (3–5 s) in a specific coating solution according to the intended dense layer thickness, making sure the whole fiber was covered. The fibers were placed such that they did not have contact in between and were left for curing at room temperature for 1–2 days. Thermal accelerated aging treatment was carried out by securely fastening the fibers in an oven and heating it up to 100 °C for 1 h for the complete crosslinking of the polymer. After, they were left for cooling at room temperature for one day. A uniform film of PDMS was formed on top of the fiber.

The fibers were potted into modules for pervaporation tests, for which epoxy adhesive (DP 105, 3M Scotch-Weld) was used to seal the ends of the module. Each module consists of 15 fibers with a length of 15 cm, and the total membrane area was 28.3 cm^2^ based on the internal diameter of the HF.

In addition, PDMS dense films were prepared to be used in other characterization tests such as contact angle measurements and to establish an indicative relationship between the viscosity of the PDMS polymer solution and the thickness of the film. A small glass plate was immersed in the same PDMS coating solution used for the second layer formation and followed by the same crosslinking process after which the film thickness was measured by using a digital micrometer (Mitutoyo, Germany).

### 2.3. Membrane Characterization

The thickness of the dense layers and cross-sectional morphologies of the composite membranes were determined by means of scanning electron microscopy (SEM, model Zeiss EVO MA15). The samples were prepared by immersing and fracturing the membranes in liquid nitrogen, followed by gold thin film deposition using a sputter coater.

The static contact angle for a PDMS film was measured by the sessile liquid drop method using a contact angle measurement system (DSA25, Krüss, Germany) in order to obtain information about hydrophobicity/hydrophilicity and the wetting behavior of the membranes prepared. For the goniometric measurements, a flat sheet membrane was prepared as described in the previous section. A 2.0 µL drop of different pure solvents (water, acetone, butanol, and ethanol) and ABE solution were deposited on the material membrane’s surface at five different sites. Each value was obtained using the software provided through image recognition. The average value for the contact angle was then considered.

Additionally, the results of TGA and FTIR analyses for the membranes used in this work were included as Supplementary Materials. The thermal stability of the components used in this work (Pebax 2533, PDMS, and PP) was examined by thermogravimetric analysis (TGA), both individually and together on the hollow fiber membrane. The TGA experiments were performed using a DTG-60H Shimadzu thermobalance. Membrane samples with an initial mass between 3 and 15 mg were placed on an alumina cell. The samples were heated up to 700 °C at a heating rate of 5 °C min^−1^ in nitrogen (25 mL min^−1^). In the specific case of PDMS, the final temperature was 1000 °C because this material has a higher resistance to degradation in a nitrogen atmosphere. On the other hand, an ATR-FTIR analysis of the polymers used for the fabrication of the selective layer of the membranes developed in this work, Pebax 2533 and PDMS (as dense homogeneous films), were carried out using a Perkin Elmer spectrum 65 Fourier Transform Infrared Spectrometer in the region of 400–3900 cm^−1^. In addition, this analysis was carried out on one of the hollow fiber membranes composed of a polypropylene support, a dense Pebax layer, and a second (outer) dense PDMS layer.

### 2.4. PV Experiments

**Figure 1:**
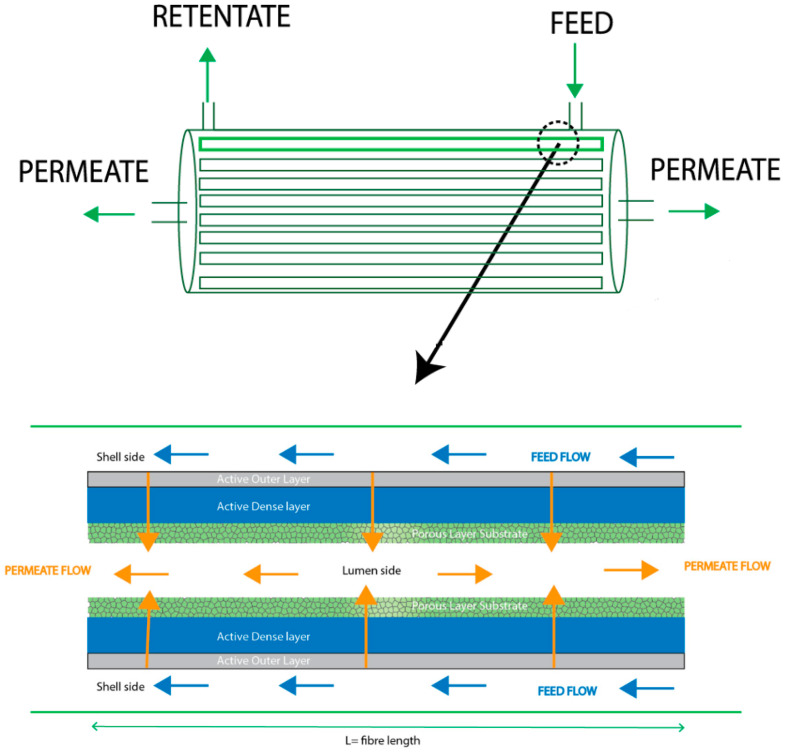
Pervaporation module.

The pervaporation experiments were carried out in a laboratory-scale unit supplied by Sulzer Chemtech (Germany) that was previously used by the authors in other pervaporation studies [37,38]. The separation process takes place when the feed mixture goes through the shell side, and the permeate comes out of the lumen side when the vacuum is applied (Figure 1). Unless otherwise stated, pervaporation tests were performed at 40 °C, since this temperature is within the appropriate temperature range to carry out the ABE fermentation process as indicated in the literature [39]. The permeate side was kept below 10 mbar using a diaphragm vacuum pump (Vacuubrand PC 3004 VARIO), and samples of the feed and permeate were collected every 30 min and were weighed and analyzed by gas chromatography (GC). After condensation, the permeate undergoes a separation of phases into an n-butanol-rich phase and a water-rich phase. After measuring the mass, the permeate was diluted with water to form a single-phase solution and then ABE concentrations were measured by GC. Samples were analyzed by duplicate in a headspace gas chromatograph (GCMS-QP2010, Ultra Shimadzu) equipped with a flame ionization detector (FID). Compounds were separated into a DB-Wax 30 m × 0.25 mm × 0.25 μm column with a detector temperature of 270 °C. Helium was used as a carrier gas at a flow rate of 82 mL min^−1^. The oven temperature was initially set at 80 °C and was subsequently increased to 150 °C at 10 °C min^−1^. GC calibration was performed with external standards. Each PV experiment lasted for at least 4 h after the stabilization process (stable operating temperature) to be sure that a pseudo steady state was reached.

The PV performance of a membrane was evaluated in terms of permeate flux *J*, membrane selectivity *α*, separation factor *β*, and pervaporation separation index (PSI). The total flux *J* (kg m^−2^ h^−1^) across the membrane is obtained by relating the mass of permeate collected with the time interval and the membrane area, as follows:

(1) $$J=\frac{m}{A_{m}\;\Delta t}$$

After that, the flux for each component *J_i_* is calculated from the total flux and the permeate composition obtained by chromatographic analysis. Membrane selectivity *α*, separation factor *β*, and the pervaporation separation index (PSI) are calculated using Equations (2)–(4), respectively:

(2) $$\alpha_{i,j}=\frac{P_{i}}{P_{j}}$$

(3) $$\beta_{i,j}=\frac{y_{i}/y_{j}}{x_{i}/x_{j}}$$

(4) $$PSI=J\;\left(\beta_{i,j}-1\right)$$

In this work, Equation (5) was adopted to describe the flux of a permeant species, where the driving force for mass transfer is a function of permeant activity and the overall resistance (*R_i,OV_*) includes contributions of resistances from the liquid boundary layer and the membrane itself, as shown in the next section.

(5) $$J_{i}=\frac{1}{R_{i,\;OV}}\left(a_{i,\;F}-a_{i,\;P}\right)$$

## 3. Results and Discussion

### 3.1. Membrane Characterization

In order to establish a frame of reference for the subsequent preparation of hollow fibers with PDMS dense protective layers with different thicknesses, we started by preparing a series of homogeneous PDMS films. As previously detailed in Section 2.2, polymer solutions with different PDMS contents were used, whose viscosity were measured using a rotational viscometer. Subsequently, the thickness of the PDMS films obtained was measured using a digital micrometer. Table 1 shows the results obtained, where it can be seen how the thickness of the films ranged from 2 to 80 μm as the concentration of the PDMS polymer solution and its viscosity increased.

**Figure 2:**
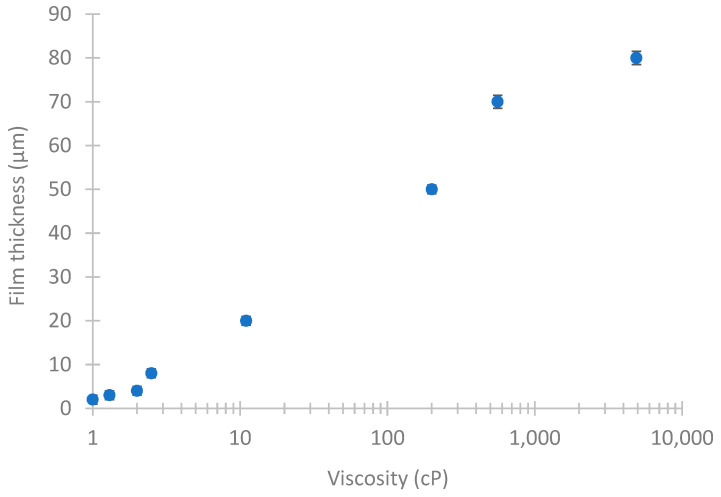
Influence of the viscosity of the PDMS polymer solution on the film thickness.

Figure 2 shows how the thicknesses of the dense PDMS films obtained by dip-coating depend on the viscosity of the polymer solution at room temperature. This trend is an indicative useful guide for selecting the characteristics of the coating solution when it is intended to obtain dense layers with a thickness less than 5 μm, especially due to the practical difficulty in handling polymer solutions with high viscosity and non-Newtonian behavior [40]. Thus, PDMS polymer solutions in the same range of viscosities shown in Figure 2 were later used as coating solutions to cover the hollow fibers with a thin outer dense layer.

In order to obtain additional information about the characteristics of the polymeric materials used, we measured the contact angle between the PDMS film and different chemical compounds that make up the ABE solution. The measured contact angles for PDMS are shown in Table 2, where the measured values for Pebax 2533 films, which were already reported in a previous work [31], were also included as a reference. The water contact angle measured for PDMS was 101°, highlighting the hydrophobic character of the surface. Data reported in previous studies show some variability, which has been attributed to surface roughness or experimental difficulties, with data ranging from 95° to 120° [41]. As Knozowska et al. [42] mentioned, the contact angle for a given material depends on the degree of crosslinking, and this also corresponds to an increase in roughness. The contact angles for organics were always <90°, while, for ABE mixture, the measured value was very close to that of water. Also included in Table 2 are the surface tension values for pure compounds (from Dortmund Data Bank), showing that the contact angle is influenced by the surface tension but does not depend solely on it but on the affinity between the components of the solution and the surface of the polymeric material. Several authors have reported the relationship between the contact angle and the surface free energy (SFE). In general, wetting and lower contact angles occur when the surface and the liquid have similar surface energies (surface tensions, in the case of the liquids). In the case of water, a lower contact angle value was observed for Pebax 2533-based membranes compared to PDMS membranes, the SFE values being in the opposite order, according to data calculated by Knozowska et al. [42].

### 3.2. Membrane Performance in Pervaporation of ABE Solutions

This section reports the results corresponding to pervaporation tests carried out with membrane modules built with different sets of hollow fibers. It was worked with various thicknesses of the dense layers in order to be able to determine the contribution of each material to the membrane performance. 

PV experiments were performed with each membrane module flowing (1:2:1 wt%) ABE solutions at 40 °C. For each experiment, the partial permeation fluxes were related to the driving force (activity gradient) to obtain the overall resistance to mass transfer (see Equation (5)). The activity coefficients for the components in the liquid feed mix were evaluated by the NRTL method using the Aspen Plus software.

It is well known that, in separation operations with selective membranes, the resistance to mass transfer in the fluid phase of the feed adjacent to the membrane can have a notable influence on the separation performance. Usually, hydrophobic membranes are significantly more permeable to dissolved organic compounds than to water, causing a depletion of the former compounds in the liquid boundary layer. This phenomenon is known as concentration polarization. Such effects depend mainly on the hydrodynamic conditions in the liquid phase and are usually investigated by changing the feed flow rate in pervaporation experiments. Therefore, it is essential to be able to quantify the incidence of the concentration polarization phenomenon in our tests in order to later be able to analyze the intrinsic resistance of the membrane. In a previous study [31] working with PEBA thin-film composite hollow fiber membranes, it was reported that the total resistance was fitted (Wilson plot) by the reciprocal of the lineal velocity u (m min^−1^) through the membrane module raised to an exponent of 0.9, a factor frequently adopted for parallel flow in membrane contactors [43,44]. In this work, further analysis of those permeation data for organic compounds was made in order to obtain a correlation that describes the transport parameters in terms of characteristic dimensionless numbers. Given the cylindrical configuration of the hollow fibers and taking into account that, in this study, it was adopted referring the permeation fluxes in all cases to the internal area of the support (PP), the contribution of the individual mass transfer resistances to the overall resistance for the case of fibers with a single selective dense layer of PEBA is given by the following equation:

(6) $$R_{i,\;ov}=\frac{1}{k_{i,\;ov}}=\left(\frac{\gamma_{i,\;F}}{k_{i,\;bl}\;\rho_{m}}\right)\times \frac{A_{supp,\;in}}{A_{fiber,\;out}}+\frac{\delta_{PEBA}}{P_{i,\;PEBA}}\times \frac{A_{supp,\;in}}{A_{LM,\;\;PEBA}}+R_{i,\;supp}$$

where *k_i,ov_* is the mass transfer coefficient for component *i*; *k_i,bl_* is the mass transfer coefficient for component *i* at the liquid boundary layer; γ*_i,F_* is the activity coefficient for component *i* in the liquid phase; *ρ_m_* is the molar density of feed liquid; *P_i,PEBA_* is the membrane permeability for component *i* through the *PEBA* layer; *δ_PEBA_* is the thickness of the *PEBA* layer; *R_i,supp_* is the mass transfer resistance in membrane support; *A_supp,in_* is the membrane area based on the internal diameter of the porous support; *A_fiber,out_* is the membrane area based on the outer diameter; *A_LM,PEBA_* is the logarithmic mean area of the *PEBA* layer.

The mass transfer coefficient at the feed boundary layer (*k_i,bl_*) depends on the circulation configuration of the ABE solution in the membrane module. In this system, the ABE solution circulates through the shell side in order to maximize mass transfer coefficients, increasing the transfer area and improving the hydraulic conditions [45]. Usually, the mass transfer coefficients in the liquid boundary layer are correlated through the Sherwood number (*Sh*) as a function of the dimensionless Reynolds (*Re*) and Schmidt (*Sc*) numbers. Numerous papers have dealt with reviewing and analyzing the correlations proposed for predicting shell-side mass-transfer. Among them, the papers by Lipnizki and Field [46], Shen et al. [47], and the recent work by Estay et al. [45] deserve to be highlighted. For the calculation of the Reynolds number, the definition of equivalent diameter (*d_eq_*) for the shell-side flow proposed by Dahuron and Cussler [48] was adopted, as follows:

(7) $$d_{eq}=\frac{4\times \left(\mathrm{cross-sectional}\;\mathrm{area}\;\mathrm{of}\;\mathrm{flow}\right)}{\mathrm{wetted}\;\mathrm{perimeter}}=\frac{d_{shell}{}^{2}-NF\times d_{out}{}^{2}}{d_{shell}+NF\times d_{out}}$$

Thus, the Reynolds number is calculated as follows:

(8) $$S_{shell}=\frac{\pi }{4}d_{shell}{}^{2}-NF\times \frac{\pi }{4}d_{out}{}^{2}$$

(9) $$u_{shell}=F/S_{shell}$$

(10) $$Re_{shell}=\frac{u_{shell}\times \rho_{mass}\times d_{eq}}{\mu }$$

The mass transfer coefficient in the liquid boundary layer (shell-side) is related to the Sherwood number as follows:

(11) $$Sh=\frac{k_{i,\;bl}\times d_{eq}}{D_{i}}$$

where the equivalent diameter is used as the characteristic length. The values of the diffusion coefficients (*D_i_*) for organic compounds in aqueous solution was estimated using the Wilke-Chang correlation.

Thus, an estimation of parameters was carried out to achieve a correlation that links the mass transfer coefficient at the liquid phase with the properties and operating conditions. For this, a set of experimental data was used that was obtained from PV tests with various modules built with hollow fibers coated with dense layers of PEBA with various thicknesses, working with ABE solutions at 40 °C and different flow rates (0.2, 0.3, 0.5, 1.2, 2.0, and 4.5 L min^−1^). Reynolds numbers for the experiments were in the range of 340–7800, and the length of the modules was 15 cm in all cases. The estimation procedure established that the correlation that best describes the mass transfer in the liquid phase circulating through the shell-side in flow parallel to the fibers is the following:

(12) $$Sh=0.025\;\left(1-\varphi \right)\;Re^{0.9}\;Sc^{0.33}$$

where the packing fraction (*φ*) was calculated as follows:

(13) $$\varphi =\frac{NF\times d_{out}{}^{2}}{d_{shell}{}^{2}}$$

**Figure 3:**
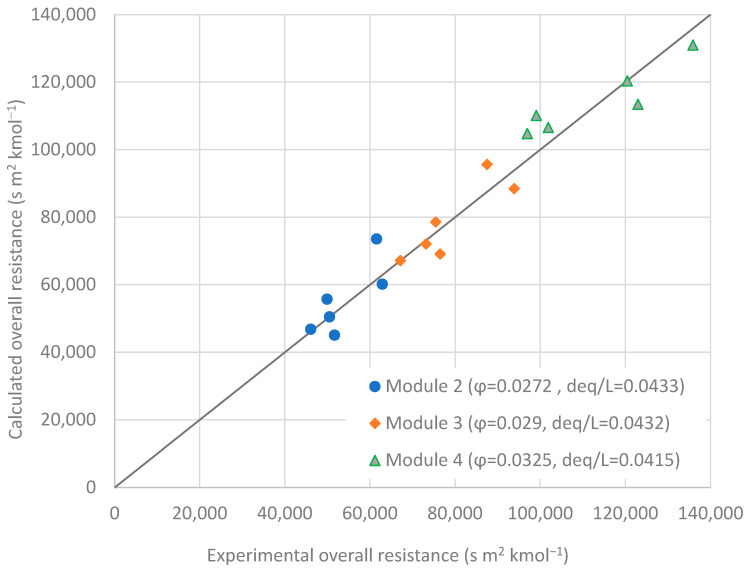
Parity graph of simulated overall resistances vs. experimental overall resistances for butanol permeation. Values were obtained and calculated for three modules at different flow rates.

The parameter estimation procedure allowed, at the same time, to determine the permeability values of PEBA and resistance in the support for each one of the permeants, whose values are shown in Table 3. In the case of water as permeant, the mass transfer resistance in the liquid phase was assumed negligible. The overall mass transfer resistance values for butanol calculated with the model for three membrane modules were plotted against the experimental data to build the model parity graph (Figure 3), proving that the fit of the model can be taken as satisfactory, with good agreement between the experimental and simulated data. The small difference in the *R_i,supp_* and *P_i,PEBA_* values reported in Table 3 with respect to those reported in previous work [31] is due to the fact that, in this work, in Equation (6) the ratio of areas in each term was included so that the mass transfer resistances were referred in all cases to the internal area of the support. These data confirm that the order in which organic compounds permeate through PEBA in terms of permeabilities (kmol m^−1^ s^−1^) is given by: *P*_BuOH_ > *P*_EtOH_ > *P*_Acet_. The intrinsic selectivity of PEBA membranes towards n-butanol is explained by the preferential sorption of n-butanol over acetone and ethanol, as reported by experimental studies by Liu and Feng [49] and Heitmann et al. [50].

Below are the results obtained in PV tests working with membrane modules made of hollow fibers consisting of two dense layers of selective material: a first dense layer of PEBA deposited on the PP support and a second dense (outer) layer of PDMS. Different thicknesses of PDMS for the outer layer were deposited by dip-coating in the range of 3–80 μm. Thus, the contribution of the individual mass transfer resistances to the overall resistance for the case of fibers with two selective dense layers is given by the following equation:

(14) $$R_{i,\;ov}=\frac{1}{k_{i,\;ov}}=\left(\frac{\gamma_{i,\;F}}{k_{i,\;bl}\;\rho_{m}}\right)\times \frac{A_{supp,\;in}}{A_{fiber,\;out}}+\frac{\delta_{PDMS}}{P_{i,\;PDMS}}\times \frac{A_{supp,\;in}}{A_{LM,\;\;PDMS}}+\frac{\delta_{PEBA}}{P_{i,\;PEBA}}\times \frac{A_{supp,\;in}}{A_{LM,\;\;PEBA}}+R_{i,\;supp}$$

where *P_i,PDMS_* is the membrane permeability for component *i* through the PDMS layer; *δ_PDMS_* is the thickness of the PDMS layer

Membrane modules were assembled with each type of fiber, which, in all cases, contained 15 fibers with a useful length of 15 cm. The PV experiments were performed at 40 °C, while the feed flowrate was 4.5 L min^−1^, which was the maximum possible flow rate for the feed liquid on the shell side in order to minimize the mass transfer resistance in the liquid boundary layer. The data collected in these experiments made it possible to evaluate the PDMS permeability values for the different permeants by means of Equation (14), taking into account that the permeabilities of PEBA were previously determined. Analyzing the PDMS permeability values for the organic compounds reported in Table 3, these follow the order acetone > n-butanol > ethanol when the driving force for mass transfer is the difference in activities for the permeant species across the membrane. Although it is not easy to make a comparison with previous studies reported in the literature due to the influence of different materials and working conditions, the higher permeability through PDMS for acetone over n-butanol and ethanol is consistent with the results presented by Rozicka et al. [51] and van Wyk et al. [52]. In this sense, Rozicka et al. [51] reported the performance of three commercial PDMS-based membranes (Pervatech, Pervap 4060 and PolyAn) in the pervaporation removal of acetone, butanol, and ethanol from binary aqueous mixtures at 25 °C. Using the data reported in that study to evaluate permeabilities (recalculating them to consider the difference in activities for the permeant species across the membrane as the driving force for mass transfer), it turns out that the highest permeability value corresponds to acetone, as well as higher separation factors for acetone over n-butanol and ethanol. Results of the same order were also reported by van Wyk et al. [52] in a study on the separation of ABE model solutions with PDMS membranes (Pervatech) in the range of 30–50 °C. The behavior of PDMS membranes more favorable to acetone permeation compared to PEBA membranes can be largely attributed to the different solvent uptake of both membrane materials. As mentioned above, the experimental studies by Liu and Feng [49] and Heitmann et al. [50] showed that the solvent uptake in PEBA 2533 is considerably higher for n-butanol than for acetone or ethanol. However, swelling studies for PDMS membranes, carried out with both pure solvents [51,53] and aqueous solutions [50], have shown that the acetone uptake is at least similar to or even higher than that of butanol, while the ethanol uptake is lower than that of the other two organics. The higher affinity of PDMS for acetone compared to the other permeants was also reported by Yang et al. [54] in a study that used inverse gas chromatography for the characterization of the solubility thermodynamics and diffusion of solvent-PDMS systems.

**Figure 4:**
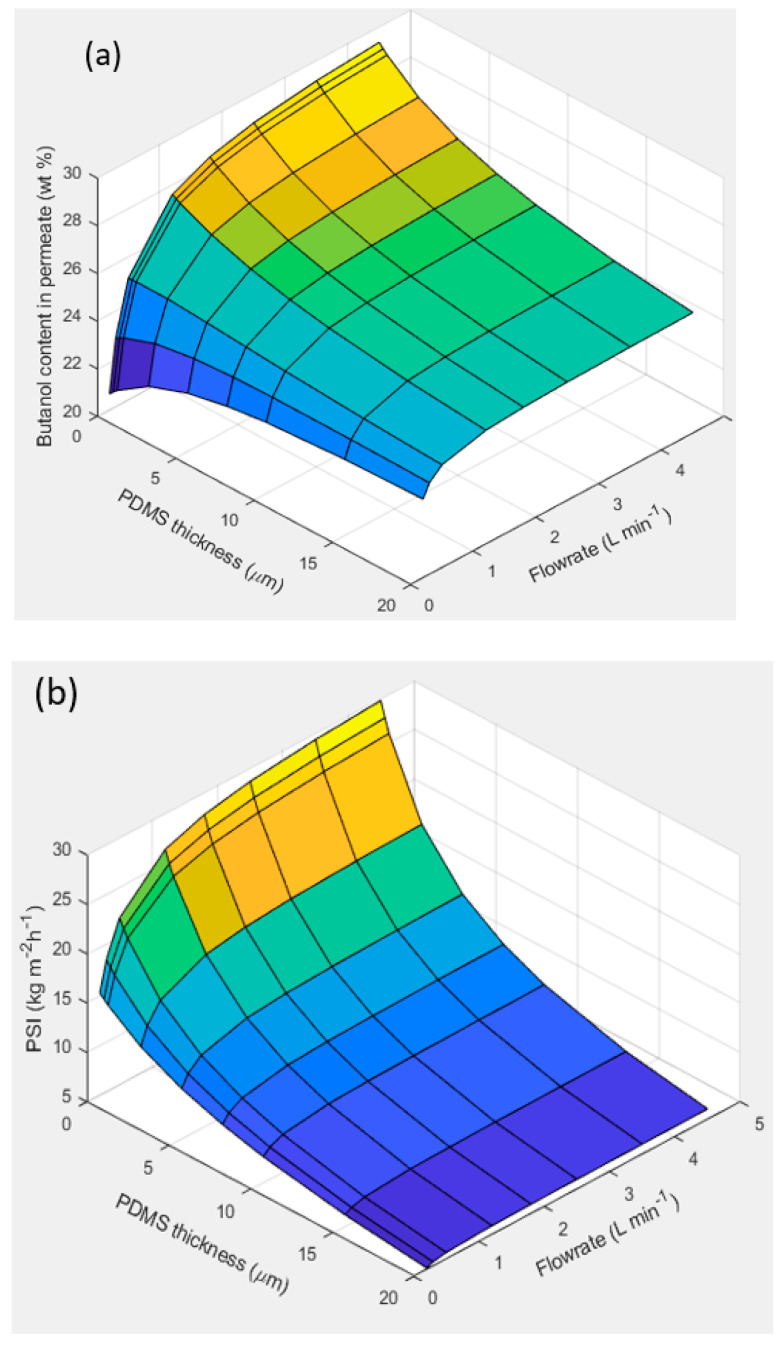
Simulation showing the influence of the feed flow rate and thickness of the PDMS dense layer on (**a**) butanol content in permeate (wt%) and (**b**) PSI, for a membrane module 1 m in length (operating conditions: 40 °C; downstream pressure: 10 mbar; feed mixture: (1:2:1 wt%) ABE solutions).

In order to have a broader view of the variables that influence the separation performance of a PV module with dual-layer hollow fibers, a sensitivity analysis performed with simulation tools is presented below. Since the fiber length is usually around 1.0–1.5 m in membrane modules for industrial applications, we performed a simulation study to evaluate the influence of the PDMS dense layer thickness and the feed flow rate on the separation performance for a hypothetical membrane module 1 m in length. In all cases, fibers that included the porous support of PP and a selective dense layer of PEBA with a thickness of 1.6 μm were considered, on which a dense protective layer of PDMS was deposited, with variable thicknesses in the range of 0 to 20 μm. The equations that describe the mass transfer in the HF membranes (Equations (5)–(14)) together with the material and energy balances were included in a distributed parameter model that was implemented in Aspen Custom Modeler, making use of Aspen Plus subroutines for the estimation of thermodynamic properties (densities, vapor pressures, activity coefficients, enthalpies) and transport properties (viscosity, diffusion coefficients), and then a series of simulations were run. Setting the separation/concentration of n-butanol as a priority, the most favorable results in terms of PSI and butanol content (wt%) in the permeate stream correspond to the hollow fibers with the thinnest thickness of the PDMS dense layer and the highest feed flow rate (4.5 L min^−1^), as shown in Figure 4. It is important to highlight the relevant effect that the fluid dynamic conditions in the liquid phase of the feed can have on the separation performance when selective membranes are used for the removal of organics from dilute solutions. Thus, considering the case of a dual-layer HF membrane with a PDMS layer thickness of 1 μm and by increasing the flow rate from 0.2 to 4.5 L min^−1^ (which corresponds to a Reynolds number interval of 340–7690), the contribution of the boundary layer resistance to the overall mass transfer resistance decreased from 39% to 3.7% for butanol, while the butanol content in the permeate increased from 21.2 to 28.7 wt%. This phenomenon is probably due to the transition from a laminar to a turbulent flow for the feed liquid on the shell-side.

From the simulations, it was observed that, as the PDMS layer becomes thicker, it becomes more difficult to remove the butanol; thus, the boundary layer resistance becomes insignificant, regardless of its magnitude. We can see that, in thicker PDMS layers, there is less variation in the content of n-butanol in the permeate with a change in the feed flowrate due to the lower contribution of the boundary layer resistance to the overall resistance. 

## 4. Conclusions

The experimental results allowed for finding a relationship between the viscosity of the PDMS coating solution and the thickness of the dense layer that can be achieved by the dip-coating procedure. The mass transport phenomena in the pervaporation process were characterized using a resistances-in-series model. Knowing that, under working conditions, fluid dynamic conditions can significantly influence the separation achieved, a correlation for the mass transfer coefficients in the liquid boundary layer as a function of the dimensionless Reynolds (Re) and Schmidt (Sc) numbers was developed. The parameter estimation procedure allowed to determine that the correlation that best describes the mass transfer in the liquid phase circulating through the shell side in the flow parallel to the fibers is $Sh=0.025\;\left(1-\varphi \right)\;Re^{0.9}\;Sc^{0.33}$ for HF modules with low packing fraction and Reynolds numbers in the range 340–7800. The PV results showed that PEBA, as the material of the dense separating layer, is the most favorable in terms of selectivity towards butanol with respect to the other organics. The addition of a protective layer of PDMS allows the sealing of possible pinholes; however, its thickness should be kept as thin as possible since permeation fluxes decrease with the increasing thickness of PDMS, and this material also shows greater selectivity towards acetone compared to other organics.

## Figures and Tables

**Table 1:** Thickness of films obtained from polymeric solutions with different PDMS contents.

PDMS (wt%) ^(a)^	Viscosity (cP)	Film Thickness (µm)
5	1 ± 0.06	2 ± 1
10	1.3 ± 0.06	3 ± 1
15	2 ± 0.06	4 ± 1
20	2.5 ± 0.06	8 ± 1
40	11 ± 0.2	20 ± 1
60	200 ± 3	50 ± 1
80	560 ± 7.5	70 ± 1.5
100	4900 ± 60	80 ± 1.5

^(a)^ It corresponds to the dilution of the two-component kit in n-hexane.

**Table 2:** Contact angle measured for selective polymers and ABE components.

Liquid	Measured Contact Angle [°]				Surface Tension (mN m^−1^), 293.15 K
	PDMS		Pebax 2533 [31]		
	Value	SD	Value	SD	
Butanol	35.7	3.8	38.4	6.2	24.6
Ethanol	42.3	4.4	55.0	8.8	22.3
Acetone	38.6	9.6	32.4	13.2	23.3
Water	101.0	3.3	76.8	8.2	72.8
ABE mixture	100.1	11.7	70.2	7.3	

SD: standard deviation.

**Table 3:** Mass transfer resistance of the support and permeability of the membrane selective layers.

Compound	Resistance in Support (m^2^ s kmol^−1^)		PEBA Permeability (kmol m^−1^ s^−1^)		$\mathrm{PEBA}\;\mathrm{Selectivity}\;\alpha_{i/water}$	PDMS Permeability (kmol m^−1^ s^−1^)		$\mathrm{PDMS}\;\mathrm{Selectivity}\;\alpha_{i/water}$
	Value	S.E.	Value	S.E.		Value	S.E.	
n-butanol	35.2 × 10^3^	2.6 × 10^3^	3.0 × 10^−10^	1.9 × 10^−11^	0.64	1.1 × 10^−10^	2.1 × 10^−11^	1.67
ethanol	42.5 × 10^3^	3.6 × 10^3^	2.0 × 10^−10^	1.1 × 10^−11^	0.41	5.3 × 10^−11^	1.8 × 10^−11^	0.80
acetone	34.6 × 10^3^	4.2 × 10^3^	7.6 × 10^−11^	3.3 × 10^−12^	0.16	3.2 × 10^−10^	3.8 × 10^−11^	4.85
water	74.2 × 10^3^	2.1 × 10^3^	4.7 × 10^−10^	3.8 × 10^−11^		6.6 × 10^−11^	2.0 × 10^−11^	

S.E.: standard error.

## Data Availability

Data is contained within the article or Supplementary Materials.

## Supplementary Materials

The following supporting information can be downloaded at: https://www.mdpi.com/article/10.3390/membranes12101007/s1, Figure S1: Influence of the PDMS content in the polymer solution on the film thickness; Figure S2: TGA of polypropylene, Pebax and PDMS polymers, and a hollow fiber membrane with two dense layers (Pebax + PDMS) on a PP support; Figure S3: Differential thermogravimetric (DTG) curves of the studied materials; Figure S4: ATR-FTIR analysis for Pebax, for PDMS and for the hollow fiber composite membrane.

## Abbreviations

The following abbreviations are used in this manuscript:

HF	hollow fiber
PDMS	poly(dimethylsiloxane)
PEBA	poly(ether-block-amide)
PP	polypropylene
PSI	Pervaporation Separation Index (kg m^−2^ h^−1^)
PV	pervaporation
Symbols and Units	
$a_{i}$	activity of component *i* (mole fraction, dimensionless)
*A*	area (m^2^)
$d_{eq}\;$	equivalent diameter (m)
$D_{i}$	diffusion coefficient for component *i* (m^2^ s^−1^)
*F*	liquid flow rate (m^3^ s^−1^)
*J*	total transmembrane flux (kg m^−2^ s^−1^)
$J_{i}$	transmembrane flux of each component (kg m^−2^ s^−1^)
$k_{i,bl}$	mass transfer coefficient for component (*i*) at the liquid boundary layer (m s^−1^)
*NF*	number of fibers in a module
$P_{i}$	permeability for component (*i*) (kmol s^−1^ m^−1^)
*Re*	Reynolds number
$R_{i,\;\;ov}$	overall mass transfer resistance (s m^2^ kmol^−1^)
$R_{i,supp}$	mass transfer resistance in membrane support (s m^2^ kmol^−1^)
*S_shell_*	shell-side cross-sectional area of flow (m^−2^)
*Sc*	Schmidt number
*Sh*	Sherwood number
*u*	velocity (m s^−1^)
$x_{i}$	mole fraction of component (*i*) in the feed stream
$y_{i}$	mole fraction of component (*i*) in the permeate stream
$\alpha_{i/j}$	selectivity (-)
$\beta_{i/j}$	separation factor (-)
$\gamma_{i}$	activity coefficient (-)
δ	thickness (m)
$\rho_{m}$	molar density of feed liquid (kmol m^−3^)
φ	packing fraction
