# Supplementary material for: PEBA/PDMS Composite Multilayer Hollow Fiber Membranes for the Selective Separation of Butanol by Pervaporation
Source: Membranes (Basel). 2022 Oct 17;12(10):1007. doi: 10.3390/membranes12101007 (PMC9610642; doi:10.3390/membranes12101007)
Supplement: Supplementary file 1 [file membranes-12-01007-s001.zip › membranes-1917264-supplementary.pdf]

PEBA/PDMS Composite Multilayer Hollow Fiber Membranes for the Selective Separation of Butanol by Pervaporation

Authors: Carla Arregoitia-Sarabia [1], Daniel González-Revuelta [1], Marcos Fallanza [1], Alfredo Ortiz [1], Daniel Gorri [1] *

(1) Departamento de Ingenierías Química y Biomolecular, Universidad de Cantabria, Av. de Los Castros s/n, 39005 Santander, Spain

Supplementary Material

- Influence of the PDMS content in the polymer solution on the film thickness
- Results of the FTIR and TGA analyzes for the membranes used in this work

Influence of the PDMS content in the polymer solution on the film thickness

Based on the data reported in Table 1, Figure S1 included below shows the thickness of the film as a function of the PDMS content, also including an equation obtained by regression that describes the relationship between these variables. This could be useful for a researcher to determine what would be the PDMS content to generate a PDMS layer with a specific thickness under the same conditions.

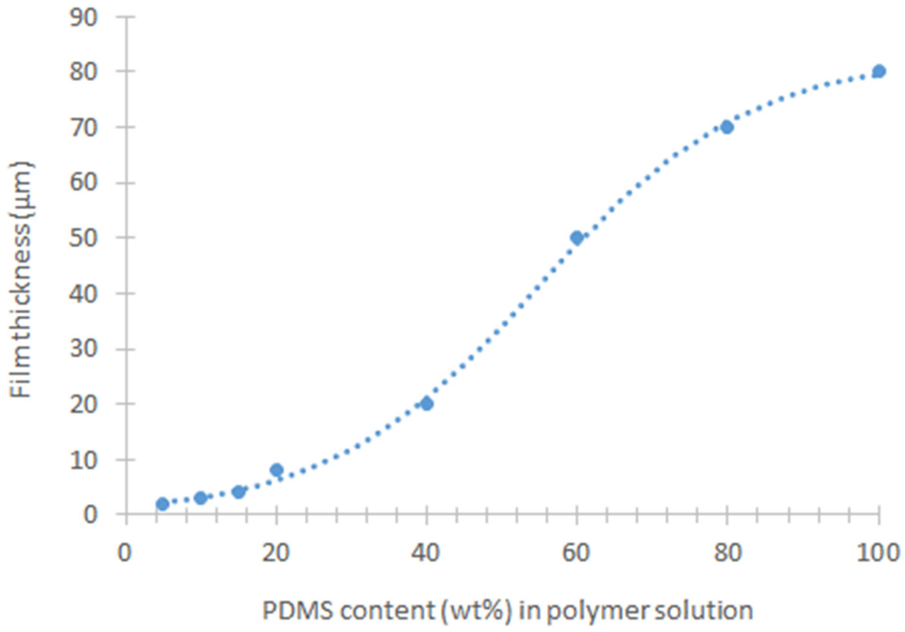

Figure S1. Influence of the PDMS content in the polymer solution on the film thickness

Fitted regression equation:

t = film thickness (µm)

$conc$ = PDMS content (wt%) in polymer solution

a, b, c = constants in regression equation

Regression equation: $t = a/(1 + b \times exp(-c \times conc))$

a = 82.6327

b = 51.04327

c = 0.07176423

Standard error: 1.195

Coefficient of determination (r^2): 0.99902

Results of the TGA and FTIR analyzes for the membranes used in this work

- **TGA analysis**

The thermal stability of the components used in this work (Pebax 2533, PDMS, and PP) was examined by thermogravimetric analysis (TGA), both individually and together on the hollow fiber membrane. The TGA experiments were performed using a DTG-60H Shimadzu thermobalance. Membrane samples with an initial mass between 3 and 15 mg were placed on an alumina cell. The samples were heated up to 700 °C at a heating rate of 5 °C min^{-1} in nitrogen (25 mL min^{-1}). In the specific case of PDMS, the final temperature was 1000 °C, because this material has a higher resistance to degradation in a nitrogen atmosphere.

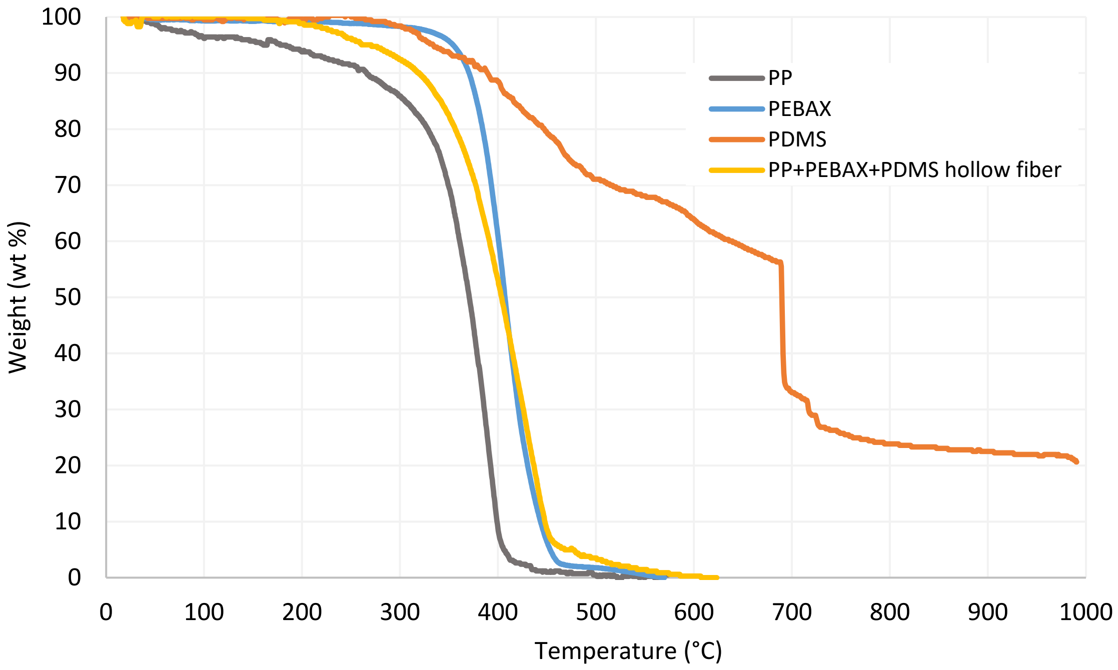

Figure S2. TGA of polypropylene, Pebax and PDMS polymers, and a hollow fiber membrane with two dense layers (Pebax + PDMS) on a PP support

As can be seen in Figure S2, the most significant weight loss in the polymers occurs at 300 °C and above. Polypropylene starts to degrade earlier than PEBAX (about 50 °C earlier), while PDMS has a higher thermal resistance. As for the hollow fiber membrane composed of the three materials, it can be seen that thermal degradation is more remarkable between 300 and 450 °C, ending up losing its mass completely at a higher

temperature due to the presence of PDMS. Additionally, Figure S3 shows the DTG curves of the materials studied.

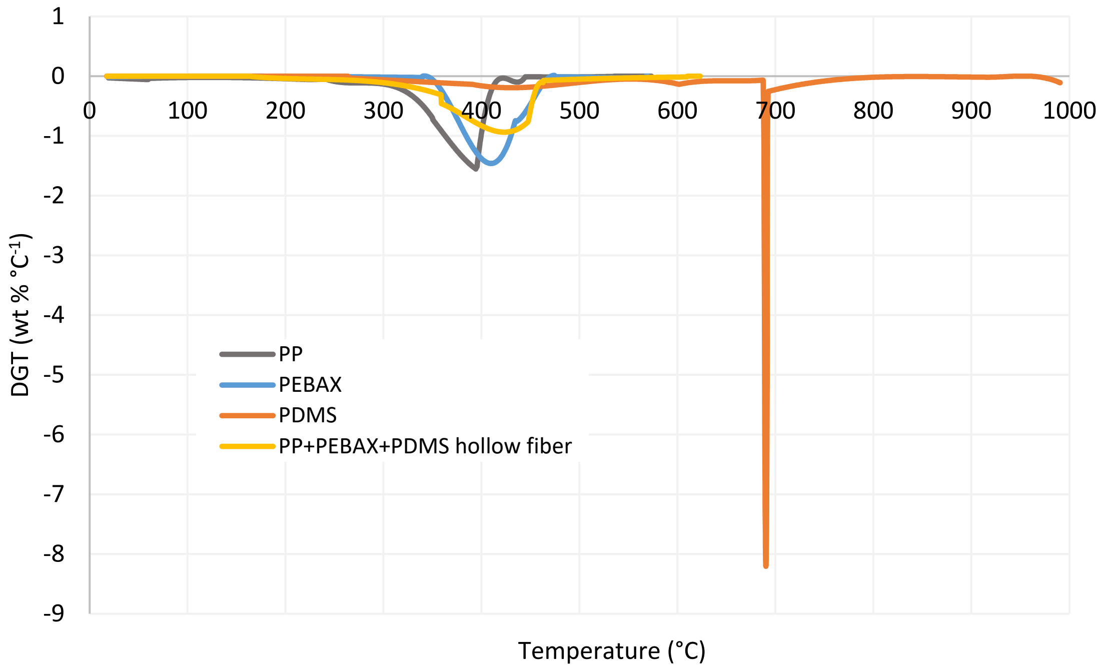

Figure S3. Differential thermogravimetric (DTG) curves of the studied materials.

- **FTIR analysis**

An ATR-FTIR analysis of the polymers used for the fabrication of the selective layer of the membranes developed in this work, Pebax 2533 and PDMS (as dense homogeneous films), has been carried out using a Perkin Elmer spectrum 65 Fourier Transform Infrared Spectrometer in the region 400-3900 cm^{-1}. In addition, this analysis has been carried out on one of the hollow fiber membranes composed of a polypropylene support, a dense Pebax layer and a second (outer) dense PDMS layer.

Figure S4 shows the results of the ATR-FTIR analysis for Pebax, for PDMS and for the hollow fiber composite membrane. As can be seen, this is a superficial technique, since the result of the analysis on the composite membrane shows the same peaks as for the PDMS, since it is the material deposited on the surface of the membrane.

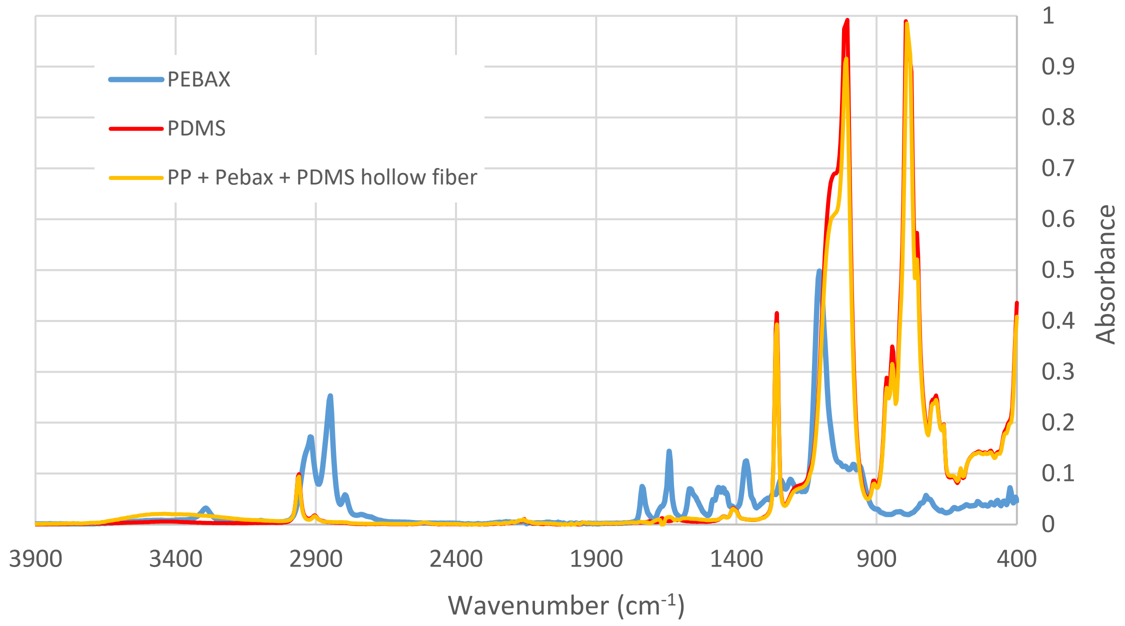

Figure S4. ATR-FTIR analysis for Pebax, for PDMS and for the hollow fiber composite membrane
